# Promiscuous antibodies characterised by their physico-chemical properties: From sequence to structure and back

**DOI:** 10.1016/j.pbiomolbio.2016.09.002

**Published:** 2017-09

**Authors:** Julie M.J. Laffy, Tihomir Dodev, Jamie A. Macpherson, Catherine Townsend, Hui Chun Lu, Deborah Dunn-Walters, Franca Fraternali

**Affiliations:** a Randall Division of Cell and Molecular Biophysics, King's College London, UK; b Department of Immunobiology, King's College London, UK; c Faculty of Health and Medical Sciences, University of Surrey, UK

**Keywords:** Antibody CDRH3, Binding promiscuity, Conformational preferences, ELISA, Kidera factors, Molecular modelling, Monte Carlo simulations

## Abstract

Human B cells produce antibodies, which bind to their cognate antigen based on distinct molecular properties of the antibody CDR loop. We have analysed a set of 10 antibodies showing a clear difference in their binding properties to a panel of antigens, resulting in two subsets of antibodies with a distinct binding phenotype. We call the observed binding multiplicity ‘promiscuous’ and selected physico-chemical CDRH3 characteristics and conformational preferences may characterise these promiscuous antibodies. To classify CDRH3 physico-chemical properties playing a role in their binding properties, we used statistical analyses of the sequences annotated by Kidera factors. To characterise structure-function requirements for antigen binding multiplicity we employed Molecular Modelling and Monte Carlo based coarse-grained simulations. The ability to predict the molecular causes of promiscuous, multi-binding behaviour would greatly improve the efficiency of the therapeutic antibody discovery process.

## 1 Introduction

Immunoglobulins (Ig) are a crucial component of the adaptive immune response. Adaptive immunity is distinct from innate immunity in that it confers a highly specific defense against invading pathogens and is capable of creating memory against foreign molecules (antigens), enabling a rapid response upon repeated exposure to the same antigen. Immunoglobulins are produced by B cells and are either displayed on the cell surface, as B cell receptors, or are secreted into the extracellular environment and circulate as antibodies in the blood.

Antigen recognition is mediated by the antibody variable regions, which are located at each of the two apical sites on the ‘Y’ arms of the antibody. A huge diversity of specificities in the antibody repertoire is achieved by gene rearrangement processes, whereby variable (V), diversity (D) and Joining (J) genes recombine to produce a complete heavy chain (VDJ) or light chain (VJ) variable gene. The rearranged gene is expressed in conjunction with a constant region that confers the functional attributes of the antibody. The heavy chain and the light chain variable regions of the immunoglobulin protein fold to form a conserved β-sheet framework interspersed by six hypervariable loops or “complementarity determining regions” (CDRs), so-called because they come together to form the antigen-binding site. Amongst the CDRs (there are three from each chain), the third loop on the heavy chain (CDR-H3) is the most diverse because it is encoded by a stretch of nucleotides spanning all three IGHV-D-J gene segments. Similarly, the equivalent light chain region (CDR-L3) is also diverse although not to the same extent. Site-directed mutagenesis and loop grafting studies have shown that the H3 loop can be sufficient to define antibody specificity (Xu and Davis, 2000). Crystal structure analyses have also identified the CDR-H3 region as being centrally positioned in the antigen-binding site, always in contact with antigen and, in some cases, able to change conformation upon binding (Stanfield and Wilson, 1994). In fact, CDR-H3 is the only exception to the canonical structure model, which has identified all remaining CDRs as belonging to one of a few discrete conformations on the basis of sequence length and composition (Shirai et al., 1996, Chothia and Lesk, 1987, Chothia et al., 1989, Shirai et al., 1999).

A consequence of the random nature of the Ig gene rearrangement process is that a significant proportion of antibodies produced in the bone marrow may be autoreactive (Wardemann et al., 2003). Potentially dangerous autoreactive antibodies must be removed from the repertoire at tolerance checkpoints in the development process in order to avoid autoimmune diseases such as systemic lupus erythematosus (Yurasov et al., 2005) and rheumatoid arthritis (Samuels et al., 2005). Some antibodies are capable of binding multiple chemically and structurally diverse antigens, which means that although an antibody may be produced with the potential to usefully bind to exogenous antigens, it may result in binding to self-antigens. Thus previous literature on tolerance and autoimmunity will quite often refer to “polyreactivity” of an antibody. Polyreactive antibodies occur in normal human sera and are thought to act as a first line of defense against foreign antigens (Guilbert et al., 1982). They have been shown to cause bacterial lysis (Zhou et al., 2007a, Zhou et al., 2007b), induce complement and clear apoptotic cells (Zhou et al., 2013). So in the case of polyreactive antibodies a trade-off balance between potentially useful initial activity and potentially harmful anti-self effects has to be maintained.

In the literature people refer indiscriminately to polyreactive and/or polyspecific antibodies and the definition intrinsic in the prefix ‘poly’ is matter of debate (Dimitrov et al., 2013). It has been recently clarified that this term does not refer to the case of multiple binding due to some stickiness of the antibody chemico-physical properties and that a large screen over a panel of putative antigens is needed before extracting antibodies that clearly react specifically to a subset of these target antigens. Another term that is frequently used when referring to antibodies multi-binding behaviour is promiscuity (James and Tawfik, 2003a, James and Tawfik, 2003b). This term has long been investigated in the field of enzyme binding (Nobeli et al., 2009), and has been referred to as functional promiscuity, again implying some specific functional behaviour resulting from the particular type of binding mode. It is claimed by Favia at al. (Nobeli et al. 2009) that the term promiscuous may imply at times what they refer to as ‘invisible’ phenotypes, observable only under certain conditions. In the past our laboratory has adopted the term promiscuity as generally applicable to proteins that show multiple partners but may use different strategies to bind in such a polyvalent way (Fornili et al., 2013). We use herewith the term promiscuous in referring to a subset of antibodies where binding to a number of tested antigens in the same experimental conditions were detected, as compared to others that do not show binding.

Despite its importance in human health and disease, the molecular basis of antibody multi-binding behaviour remains largely obscure. Towards this goal, it has been noted that some polyreactive antibodies have particularly high frequencies of aromatic amino acids in the CDR-H3 region (Droupadi et al., 1994) and high isoelectric points in heavy chain Fv regions. It has also been hypothesised that exposed hydrophobic patches are associated with so-called antibody promiscuity (Barbas et al., 1997). Somewhat paradoxically however, a role for specific hydrogen bonding has also been proposed (James and Tawfik, 2003a). Comparisons of germline versus antigen-induced antibodies have shown that the former are more likely to be polyreactive (Chen et al., 1991) and more flexible (Manivel et al., 2000), which may suggest that one of the features of polyreactive antibodies is enhanced flexibility. In support of this, a structural analysis of a promiscuous antibody in the free and bound states has demonstrated that it adopts a different conformation when bound (James et al., 2003). In contrast however, Sethi et al. (2006) have more recently shown that structurally diverse epitopes (the precise binding site on the antigen) bind differentially to a structurally common paratope (the precise binding site on the antibody), implying that paratope flexibility can be limited.

Here, we compare the structural properties of ten antibodies, four of which showed a promiscuous phenotype in enzyme linked immunosorbent assay (ELISA) and six that did not show any binding to the antigens tested. Since the encoding sequences had quite similar characteristics, we wanted to investigate the nature of the promiscuous phenotype and whether the structural properties of the antibody recognition regions were very different in these two datasets.

Using computational methods of Molecular Modelling, Kidera factor clustering and Monte Carlo conformational sampling, we identified physico-chemical properties of the CDR-H3 that are capable of distinguishing between the two phenotypes of antibody presented in this study.

## 2 Materials and methods

### 2.1 Identification of antibody heavy and light chain variable region sequences for cloning

The heavy and light chain variable region sequences for the ten antibodies used in this study are shown in Supplementary Materials Table S1. The heavy and light chain sequences had been selected, as part of another study on ageing, from datasets containing unpaired heavy and light chain variable region sequences. The datasets from which the sequences were selected are available at http://www.bcell.org.uk. As there was no information on the natural heavy-light chain pairings, the selected pairing was guided by known paired sequences in the literature (DeKosky et al., 2015).

### 2.2 Synthesis of antibodies

The chosen antibody heavy and light chain variable region nucleotide sequences were synthesised by Integrated DNA Technologies (Iowa, USA). The variable region sequences were cloned into heavy (encoding human IgG1 constant region) or light (encoding human kappa or lambda constant region) chain expression vectors using Polymerase Incomplete Primer Extension (PIPE) cloning as described previously (Dodev et al., 2014). Plasmids were then transfected into Adherent FreeStyleTM 293-F cells (Life Technologies) using FuGENE HD (Promega) according to the manufacturer's instructions. The transfected cells were maintained at 70–80% confluence at 37 °C, 5% CO_2_ in DMEM GlutaMax (31966; Gibco) supplemented with 10% (v/v) fetal calf serum (FCS) (Gibco), penicillin (5000 U/mL, Life Technologies), streptomycin (100 μg/mL, Life Technologies) and hygromycin-B (50 μg/ml, Life Technologies). Antibody-containing tissue culture supernatants were collected seven days post-transfection. The supernatants were centrifuged at 1000 g for 15 min, filtered (0.22 μm filters, Sartorius) and stored at 4 °C until use. Antibody concentration was determined by ELISA using a standard curve generated from an antibody of known concentration, as set out in Tiller et al. (2008).

### 2.3 ELISA

Antibodies in tissue culture supernatant were diluted to 1 μg/ml in PBS and were tested in triplicate against the following antigens: dsDNA (D1501, Sigma), lipopolysaccharide (LPS) (L7770, Sigma), insulin (91077C, SAFC Biosciences) and HEp-2 cell lysate (ABIN964023, Antibodies-online). Antibodies were also tested in wells that had been coated with PBS only. The choice of antigens was based on those commonly used in the literature (Wardemann et al., 2003, Tiller et al., 2008).

The ELISA protocol was as set out in Tiller et al. (2008) except for the assay development. Assays were developed by adding 50 μl OPD substrate (5 mg OPD tablet (P6912-50 TAB, Sigma) dissolved in 10 ml 1× peroxide substrate buffer (34062, Thermo Scientific) and incubating in the dark for 30 min. The reaction was stopped using 50 μl 3 M HCl. The optical density was read at 492 nm (OD_492_). A result was deemed positive if the mean OD_492_ was significantly higher than the background (i.e. secondary antibody only).

### 2.4 Antibody structures

Two structural datasets were built to analyse the distribution of CDR-H3 lengths in antibodies from all species and from humans, respectively. The “Non-redundant CDR search” option in SAbDab (Structural Antibody Database; accessible at <http://opig.stats.ox.ac.uk/webapps/sabdab>) (Dunbar et al., 2014) was used to include only complete CDR-H3 regions from crystal structures solved at a resolution of ≤ 3 Å. CDR-H3 lengths were determined according to the Chothia definition. The PDB codes for both datasets are available in the supplementary material (Supplementary Materials Table S2).

### 2.5 Antibody modelling

**Fig. 1:**
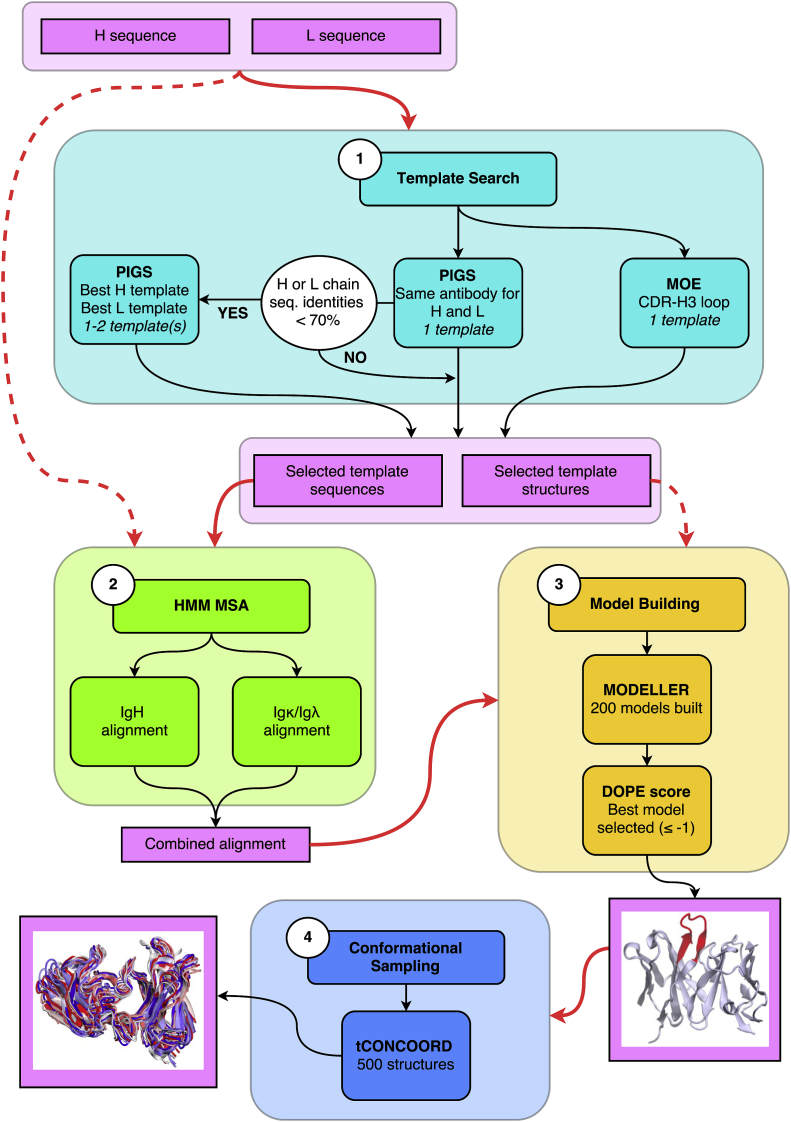
Modelling workflow. The pipeline takes the Fv regions of partner heavy and light chain sequences as input in Step 1 (cyan). The search for templates is conducted using the PIGS web server and the Antibody Homology suite in MOE. A CDR-H3-specific template is extracted from the MOE search. The PIGS “Same antibody” method is used to search for a template whose heavy (H) and light (L) chains are the best combined match for the query H and L sequences. If the sequence identity match of either chain in the PIGS template is below 70%, an additional chain-specific template is selected using the alternative “Best H and L chain” PIGS strategy. Together with the query H and L sequences, the selected template sequences are used as input in Step 2 (green). Heavy chain (IgH) and light chain (Igκ/Igλ) alignments are generated independently using isotype-specific HMM profiles developed by Tramontano and coworkers, as first described in Chailyan et al. (2011). The combined alignment and the template structures identified in Step 1 are used in Step 3 (orange). 200 models are built using the comparative modelling procedure in MODELLER and the best model is selected using normalised DOPE scoring as the quality measure. In the last step (blue), the model is used as the starting structure for tCONCOORD simulations. An ensemble of 500 tCONCOORD structures is generated using default parameters. Pink, input/output; solid red arrow, proceed to the next step in the scheme; dashed red arrow, proceed to a non-immediate step in the scheme.

The modelling workflow is described in Fig. 1 and the steps are outlined below.

#### 2.5.1 Step 1: template search

The search for templates was carried out using the PIGS web server (Prediction of ImmunoGlobulin Structure; available at <http://www.biocomputing.it/pigs>) (Marcatili et al., 2008, Marcatili et al., 2014) and MOE (Molecular Operating Environment; Version 2013.08; Chemical Computing Group, Montreal, Quebec, Canada). Both programs are considered to be state of the art in antibody modelling but differ in their methodologies (Almagro et al., 2011, Almagro et al., 2014) PIGS employs the “canonical structure” (CS) approach, which rests on knowledge that five out of the six CDRs adopt a limited number of conformations (so-called canonical structures) whose sequence determinants have been identified (Chothia and Lesk, 1987, Chothia et al., 1989). The exception to this, however, is the CDR-H3, whose sequence length and composition have so far proven too variable for complete CS rules to be identified (Shirai et al., 1996, Shirai et al., 1999, Kim et al., 1999, Morea et al., 1998, Shirai et al., 1998). Indeed, matching CDR-H3 conformations becomes increasingly difficult for cases in which the loop is particularly long. In some cases, PIGS was found to perform less when CDR-H3 sequences were queried, as previously pointed out (Marcatili et al., 2008, Marcatili et al., 2014, Marcatili et al., 2013). Furthermore, and most important in our case, PIGS does not allow the user to search for templates specific to one CDR region. For these reasons, we used instead the Antibody Homology Modelling application suite in MOE to search for a CDR-H3 specific template.

#### 2.5.2 Step 2: HMM multiple sequence alignment

The template and query sequences provide the input for Step 2: HMM Multiple Sequence Alignment. Heavy chain (IgH) and light chain (Igκ/Igλ) alignments are generated independently using isotype-specific HMM profiles developed by Tramontano and coworkers, as described in Marcatili et al. (2014). Using this alignment method, gaps in the sequence are added outwards from the centre of the CDRs and conserved residues are always aligned.

#### 2.5.3 Step 3: model building and selection

Step 3 requires the combined H-L alignment from Step 2 and the template structures retrieved in Step 1. 200 structural models are built using the comparative modelling procedure in MODELLER (Sali and Blundell, 1993) and the best model is selected using normalised DOPE scoring as the quality measure. Models with scores above −1 indicate the presence of nonnative interactions and were not considered. Models were visualised with Visual Molecular Dynamics (VMD; <http://www.ks.uiuc.edu/>) (Humphrey et al., 1996).

#### 2.5.4 Step 4: conformational sampling

In the last step, the previously selected best model is energy-minimised using the OPLS-AA force field (Robertson et al., 2015) and then used as the starting structure for tCONCOORD simulations (Seeliger et al., 2007, Seeliger and De Groot, 2009). From this, an ensemble of 500 tCONCOORD structures is generated using default parameters. We used this procedure, analogously to our previous work (Fornili et al., 2013) because this method efficiently samples the conformational space (see Fig. S6 for root-mean square distribution) and is therefore suited to antibody loop sampling, otherwise very difficult to tackle with molecular dynamics or any other atomistic-based sampling. This method is not affected by convergence issues, and has been demonstrated to extract representatives of the structural variability of proteins on the basis of both MD simulations and experimental data (Fornili et al., 2013).

In essence, tCONCOORD samples alternative conformational states *in-vacuo* by fulfilling a set of geometrical constraints extracted from the initial coordinates and interaction types (e.g., covalent bonds, hydrogen bonds, salt bridges, or hydrophobic interactions).

The tCONCOORD constraint definition is based on a statistical analysis of high-resolution X-ray structures and includes a ‘solvation score’ measure of hydrogen bond stability (Seeliger et al., 2007). Based on this description the structure of the protein is rebuilt many hundreds of times, leading to an ensemble that can be analysed.

### 2.6 Clustering analysis

A hierarchical clustering method was employed using the hclust function in R (v3.2.3) and a Minkowski distance, p, of 4. Antibodies were clustered according to the Kidera factors of their CDR-3 region amino acids (Chothia definition). The Kidera factors have been derived to encode 188 physical properties of the 20 amino acids using dimension reduction techniques. They consist of a 10-dimensional vector of orthogonal factors for each amino acid (Nakai et al., 1988).

### 2.7 Secondary structure analysis

Models were first protonated using MOE. Secondary structure predictions were performed with DSSP (Kabsch and Sander, 1983) and the output was recorded as follows: extended β-strands and β-bridges as “Beta”; α-helices, 3_10_-helices and π-helices as “Helix”; 3, 4, and 5 turns and non-hydrogen bonded bends as “Turn”; random coil as “Coil”. Normalised secondary structure probabilities were calculated for the CDR-H3 and CDR-L3 (Chothia definition).

The 95% confidence intervals were calculated using a bootstrapping method (*boot()* function in R) to generate 100 randomly resampled subsets for each of the reference datasets. This allowed any biases resulting from dataset selection to be avoided.

### 2.8 Conformational analysis

The maximally correlated motion or MCM has been calculated for the tCONCOORD trajectories. This is usually expressed as a linear combination of principal components (PCs) derived from a principal component analysis (PCA) of the system trajectory. We considered here only the first 2 PCA eigenvector components of the trajectory as these are contributing mostly to the total Eigenvalue displacement (in nm^2^) (Supplementary Materials Fig. S3). To identify the CDR-3 atoms collective motion, which maximally correlate with these two PCA components we extracted the projection of the CDR-3 atoms trajectory onto the 1st and the 2 nd PCs.

Models are available upon request to the authors' e-mails:

Julie MJ Laffy: julie.laffy@kcl.ac.uk;

Franca Fraternali: franca.fraternali@kcl.ac.uk.

## 3 Results and discussion

### 3.1 Identification of promiscuous antibodies

**Fig. 2:**
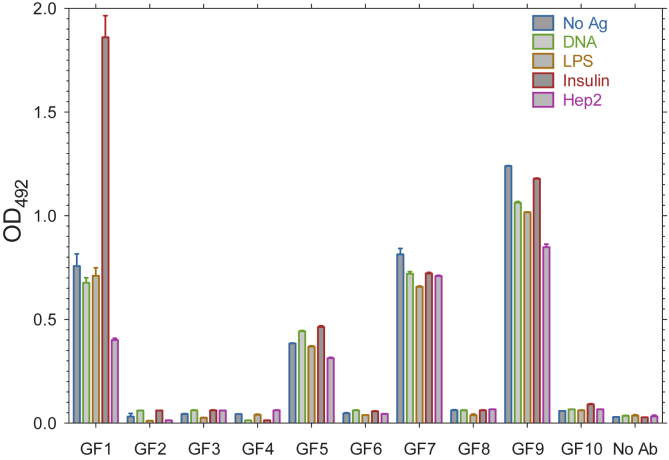
Promiscuous antibodies identified by ELISA. Ten candidate promiscuous antibodies were screened for binding against DNA, LPS, insulin and HEp2. Four of these antibodies (GF1, GF5, GF7 and GF9) gave positive results in all wells. The remaining six antibodies (GF2, GF3, GF4, GF6, GF8, GF10) produced only negative results. All wells contained phosphate-buffered saline (PBS) solution with Tween (a non-ionic detergent) acting as a blocking agent. The colour key represents the well coating used. No Ab, no antibody; No Ag, no antigen.

Ten antibodies were tested in an ELISA against a panel of four different antigens and blank, uncoated wells. It was found that the ten antibodies could be classed as showing one of the two following phenotypes: (i) a negative signal in all the ELISA wells (GF2, GF3, GF4, GF6, GF8, GF10 in Fig. 2) and (ii) a positive signal in all the ELISA wells (GF1, GF5, GF7, GF9 in Fig. 2). We named the antibodies which showed a positive signal under every ELISA condition in which they were tested “promiscuous”. Multiple sequence alignments of the promiscuous and non-promiscuous antibodies are shown in Fig. S1.

We note that the promiscuous antibodies showed a positive signal even in the absence of antigen on the ELISA plate. The reactive nature of a promiscuous class of antibodies could be such that one might expect their binding to empty wells in addition to other antigens. Nevertheless, the promiscuous antibodies clearly possess a different phenotype to the non-promiscuous antibodies, which can be detected in this ELISA format, and are relevant to previous literature where these methods have been extensively used to assess polyreactivity/autoreactivity (Wardemann et al., 2003, Tiller et al., 2008, Meffre et al., 2004). We therefore investigated this phenotype further using computational methods.

### 3.2 CDR-H3 loop length and sequence composition

**Fig. 3:**
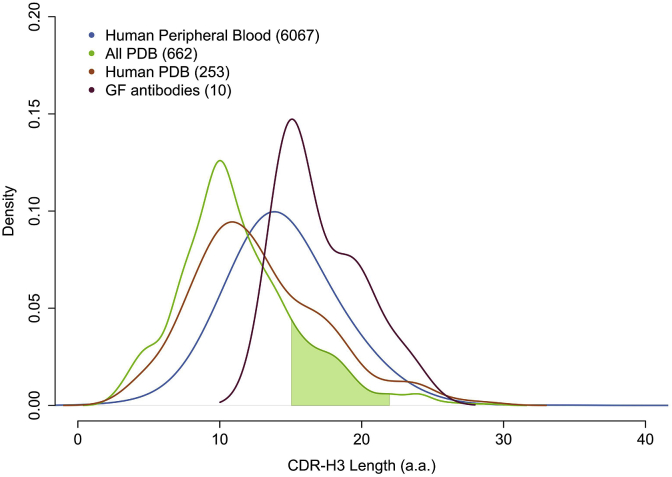
CDR-H3 length distributions. Green, antibody PDB structures from all species; Orange, Human antibody PDB structures. Blue, Human sequences from the peripheral blood (data from Wu et al. (Wu et al., 2012)); Pink, selected GF sequences. The shaded green area represents the range of templates' CDR-H3 lengths used in the structural modelling of GF antibodies. A CDR-H3 length cutoff threshold of more than 40 amino acids was applied to the PDB set containing all species.

Numerous previous studies have shown that the CDR-H3 loop is much more variable in its sequence length compared to that of the remaining CDRs, and that this is a critical determinant of its conformation. Fig. 3 shows the distribution of CDR-H3 lengths of the ten antibodies investigated in this paper (pink), a human antibody repertoire (blue) (data from Wu et al. (2012)) and antibody structures deposited in the PDB (orange, human; green, all species).

The CDR-H3 sequence length distribution of the GF antibodies analysed in this study were found to be longer, on average, when compared to both the human sequence repertoire and the PDB. Previous work has associated longer CDR-H3 regions with antibody binding multiplicity, which may render this observation significant [ref]. It should be noted however that there is no significant difference in length between the promiscuous and non-promiscuous GF subsets analysed here.

The PDB length distributions displayed a strong preference for shorter CDR-H3 loops. This may be due to the complications associated with structure determination of long, flexible regions of polypeptide. In any case, the discrepancy in CDR-H3 length between the GF antibodies and the PDB structures meant that our template search space was confined to the subset of adequate CDR-H3 lengths illustrated by the shaded green region in Fig. 3.

### 3.3 Kidera factors and β-sheet propensities are good indicators of GF promiscuity

Attempts to classify promiscuous antibodies from sequence information alone have generally been unsuccessful (see review by Dimitrov et al. (2013)). Although some studies have identified the CDR-H3 region as being accountable for the promiscuous nature of antibodies (Ditzel et al., 1996, Martin et al., 1994, Ichiyoshi and Casali, 1994), this region is always critical in antigen recognition, regardless of promiscuous behaviour.

With this in mind, we first sought to quantify the sequence-based similarities amongst the GF antibodies using a hierarchical clustering method (see Materials and Methods) and postulated that the inclusion of implicit structural information might improve the quality of our clustering analysis. As such, for each of the ten GF antibodies, protein sequences within the CDR-H3 and -L3 loops were assigned Kidera factors (a set of scores, which quantify physicochemical properties of protein sequences (see Materials and Methods) (Nakai et al., 1988).

The CDR-L3 loop was considered in this analysis, in addition to the –H3 loop, because of its demonstrable function in conferring antibody-antigen specificity. The assignment of Kidera factors to the two sequence regions were combined to give a score for each of the ten antibodies, providing a set of scores by which to cluster.

**Fig. 4:**
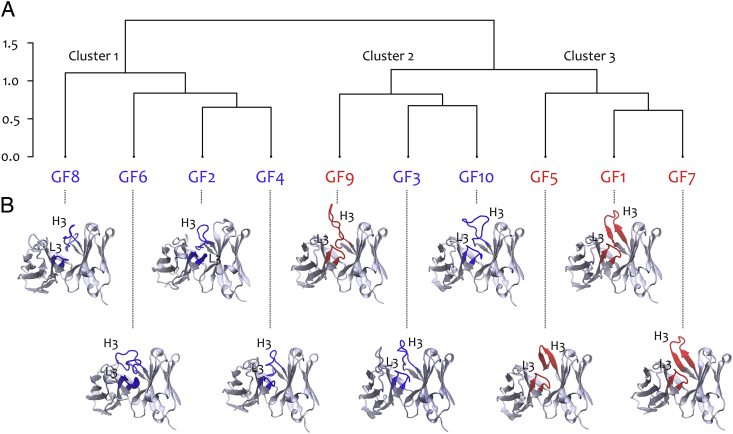
Promiscuous (red) and non-promiscuous (blue) GF antibodies can be distinguished on the basis of (A) CDR-3 Kidera properties and (B) CDR-H3 β-sheet occupancy. (A) Each node on the tree is a GF antibody represented by the Kidera factors of its CDR-3 regions. Data were clustered using the Minkowski metric with a distance *p* of 4. (B) Structural Fv models of the GF antibodies. The CDR-3 regions on the heavy and light chains are highlighted in red and blue for promiscuous and non-promiscuous cases, respectively.

Three principal clusters were defined (Fig. 4A) from this clustering procedure using Minkowski distance as the metric (see Materials and Methods). Of these clusters, two were comprised exclusively of either promiscuous or non-promiscuous antibodies. More specifically, the largest cluster (Cluster 1) contained 66.7% of the total number of non-promiscuous antibodies and 0% of the promiscuous antibodies while Cluster 3 contained 75% of the promiscuous antibodies and 0% of the non-promiscuous antibodies. Cluster 2, on the other hand, contained a mixture of both classes (25% of promiscuous cases and 33.3% of non-promiscuous cases). Promiscuous GF1 and GF7 and non-promiscuous GF2 and GF4 were the most rooted in their respective clusters, which suggested that the traits associated with either binding phenotype should be best represented by these antibodies.

It is worth noting that similar clustering analyses were performed using simpler parameters to describe the CDR-3 regions, such as hydrophobicity and charge, but no clear separation between the two sets was observed (data not shown). It can therefore be concluded that Kidera Factors are a powerful means of incorporating general physico-chemical properties and structural information into a sequence-based analysis. Interestingly, in the context of blood repertoire, similar conclusions were reached in a study by Epstein et al. (Epstein et al., 2014), which used Kidera factors to analyse TCR repertoires from different individuals.

### 3.4 Enriched β-sheet content in promiscuous CDR-H3 regions

The variable regions of each of the GF antibodies were modelled to look for explicit structural differences between members of the different clusters (see Fig. 4). In each case, the most native-like representative from a sample of 200 models was selected (presented in Fig. 4B) (see Materials and Methods for more information).

Intriguingly, the β-sheet content of the CDR-H3 was highly enriched in the promiscuous antibody models, particularly in those within Cluster 3 (Fig. 4). Moreover, the only promiscuous antibody not belonging to Cluster 3 (GF9) was also the only one that appeared to lack the ordered β-sheet seen in the other cases. In fact, the CDR-H3 secondary structure of GF9 much more closely resembled a random coil; a feature that was shared amongst all antibodies in Clusters 1 and 2.

**Fig. 5:**
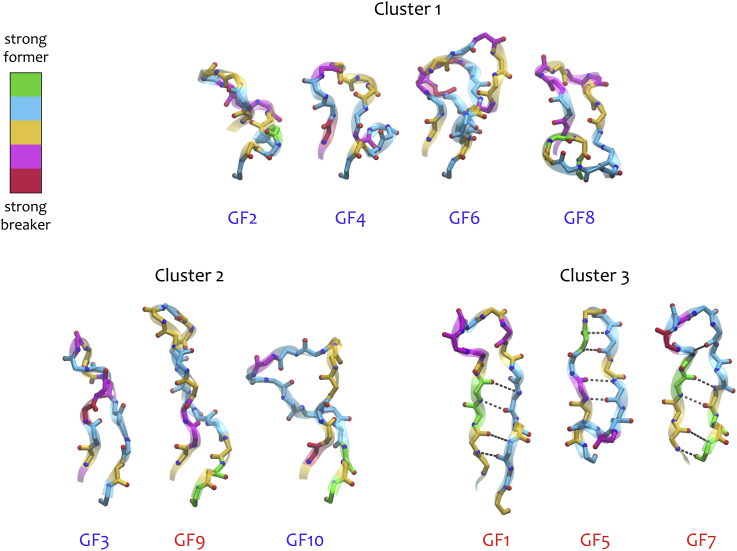
β-sheet propensities and structure of CDR-H3 residues. Backbone representation of the CDR-H3 with amino acids coloured according to their propensity to form β-sheet (Prevelige, 1989): green, strong formers (V,I,M); blue, formers (F,Y,C,T,W,L,Q); yellow, indifferent (R,G,A,D); pink, breakers (H,S,K,N,P); red, strong breakers (E). Clusters were defined according to the result in Fig. 4. GF labels in red denote promiscuous CDR-H3s; blue labels denote non-promiscuous CDR-H3s.

The β-sheet-forming propensities (Prevelige, 1989) of residues in the CDR-H3 regions (Fig. 5) also reflected the results in Fig. 4. Within Cluster 3, β-sheet breakers (pink) and strong β-sheet breakers (red) were concentrated at the apices of the CDR-H3s and were largely absent from their stems, thereby permitting anti-parallel β-sheet hydrogen bonding. The exception to this was a serine residue in GF5 (breaker; pink), however its geometry and neighbouring environment was such that a hydrogen bond still formed. Contrastingly, the distributions of β-sheet breakers were far more scattered in members of Clusters 1 and 2. A higher occurrence of strong β-sheet formers (green) in the stems of Cluster 3 members was also noted. Once again, this observation was less relevant in GF5. To this point, it is perhaps worth noting that GF5 was the least deep-seated in Cluster 3 (Fig. 4).

**Fig. 6:**
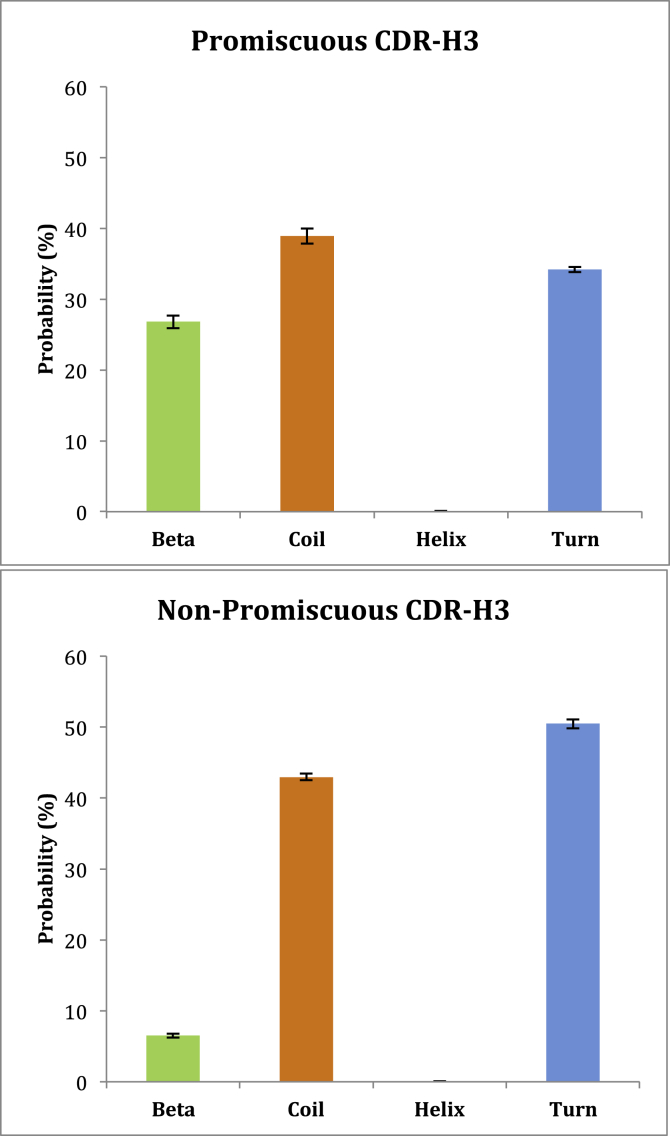
Average secondary structure probabilities in simulation ensembles for i) promiscuous and ii) non-promiscuous CDR-H3 regions. Predictions were calculated using DSSP. Each antibody is represented by a conformational ensemble of 500 tCONCOORD structures, such that the total number of structures in i) is 2000 (4 antibodies) and in ii) is 3000 (6 antibodies). The error bars represent the confidence intervals at the 95% level, estimated using 100 runs of bootstrap resampling.

Beyond β-sheet propensities, the degree of CDR-H3 collapse seemed to correlate well with membership to a particular cluster (Fig. 5). Cluster 1 CDR-H3 members were the most ‘tangled’; Cluster 2 members were comparatively taut and Cluster 3 members were most extended (forming β-sheet structure) (see Fig. 6).

To assess the statistical significance of this finding and to ensure that differences in secondary structure content were not simply a reflection of the templates used, Monte Carlo-based simulations were run on all GF antibodies using the tCONCOORD program (see Materials and Methods). CDR-H3 secondary structure probabilities were calculated for the GF ensembles of 500 tCONCOORD structures and subsequently averaged over the promiscuous and non-promiscuous sets.

As illustrated in Fig. 5, the CDR-H3 β-sheet content was 33.4% higher in the promiscuous antibodies compared to the non-promiscuous antibodies (two-tailed P value < 0.0001) and this difference was even more pronounced when GF9 was excluded from the promiscuous set (data not shown). The non-promiscuous antibodies were also predicted to have 18% more ‘turn’ secondary structure (defined as 3, 4, and 5 turns and non-hydrogen bonded bends) (two-tailed P value < 0.0001).

Predicted secondary structure occupancies were also calculated for the CDR-L3 regions of the promiscuous and non-promiscuous antibodies however no significant differences were observed (Supplementary Materials Fig. S2).

### 3.5 Key residues in the CDR-H3 of GF1

**Fig. 7:**
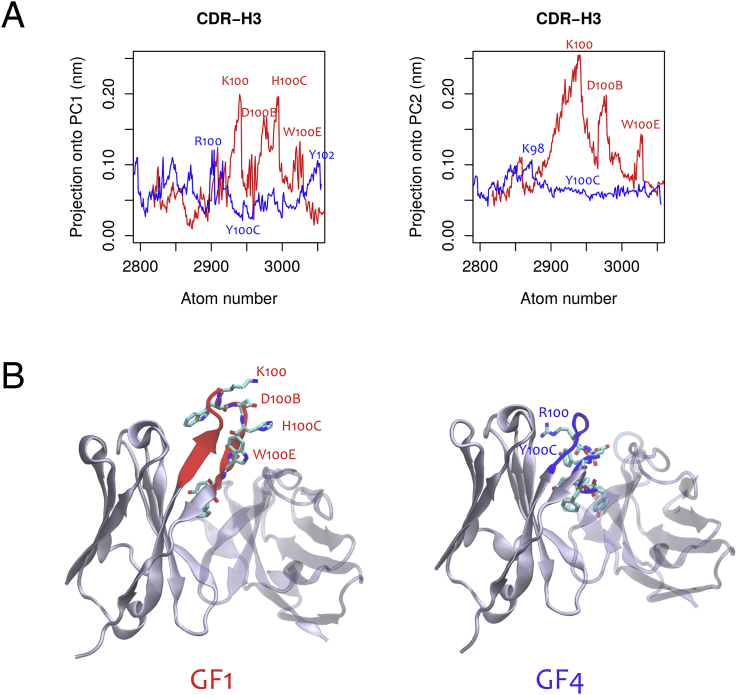
Principal component analysis of CDR-H3 dynamics. (A) The contribution of each atom in the CDR-H3 loop of GF1 (red) and GF4 (blue) to the first and second eigenvectors measured in nm^2^. (B) The residues whose atoms contributed most significantly to the respective PCs in GF1 were labelled in (A) and mapped onto the GF1 model. Corresponding residues were mapped onto the GF4 model for reference.

To reduce the high dimensionality of the tCONCOORD trajectories and to identify the dominant molecular motions related to either promiscuous or non-promiscuous antibodies, principal component analysis (PCA) was applied. Two representative tCONCOORD trajectories were taken from each class; GF1 for the promiscuous subset and GF4 for the non-promiscuous subset. As mentioned earlier in the text, these antibodies were amongst the most deep-rooted in their respective clusters (Fig. 4A). Furthermore, their CDR-H3 and CDR-L3 loops were determined to be of approximately the same length and of very high sequence similarity (CDR-H3, 99%; CDR-L3, 95%). In the trajectories of both GF1 and GF4 the dominant global motion was found to be a side-to-side ‘rocking’ of the heavy and light chain framework regions (Supplementary Materials Fig. S4). When the projection of atoms in the CDR-H3 and -L3 loops onto PC1 and PC2 were determined, several residues in the CDR-H3 loops of the promiscuous GF1 antibody, but not GF4, were also found to contribute to the global motion accounted for by the first and second eigenvectors (Fig. 7). The CDR-L3 regions were found not to contribute significantly to the global motion in either the first or the second eigenvectors (Supplementary Materials Fig. S5).

Analysis of PC1 and PC2 highlighted four particularly dynamic residues in the CDR-H3 of GF1: Lys100, Asp100B, His100C and Trp100E (Chothia numbering definition; Fig. 7A). Interestingly, these residues are concentrated at the apex of the CDR-H3 β-sheet (Fig. 7B). Furthermore, some of these residues contain features that are indicative of binding versatility. Firstly, residues such as Lys and Trp are often found at binding interfaces due to their ability to adapt to both hydrophobic and hydrophilic environments (Mian et al., 1991). Residues with a large surface area (Lys, His, Trp) should also contribute favourably to binding free energies with antigen due to their participation in van der Waals' interactions (Chothia and Lesk, 1987, Chothia et al., 1989, Gelles and Klapper, 1978). Lastly, some residues, including Tyr and Trp, are intrinsically flexible (Mian et al., 1991); a property that has previously been found as characteristic of promiscuous residues in hub proteins (Fornili et al., 2013). Such residues could sample a wider range of dihedral angles and adjust their fit to accommodate different antigens.

### 3.6 Putative binding mechanisms

We hypothesize that the advantage of a protruding β-sheet-rich CDR-H3 in the promiscuous antibodies lies in the inability of its residues to partake in intra-molecular interactions. Instead, such outwardly positioned residues would be most effectively placed to encourage interactions with foreign molecules. In contrast, the lower occurrence of β-sheet in the non-promiscuous CDR-H3 loops would result in a higher number of ‘rigidifying’ intra-molecular CDR-H3 contacts. This could explain why the CDR-H3 residues in GF4 were less dynamic than their counterparts in GF1 despite residing in an otherwise flexible random coil (Fig. 7).

Beyond this, the findings presented here suggest that specific residues in the GF antibodies confer promiscuity, as was found in a large-scale analysis of multi-binding proteins (Fornili et al., 2013). The dominant nature of the promiscuous CDR-H3 could permit the exposure of local and chemically versatile neighbourhoods of amino acids with configurations free of the steric or structural constraints normally expected within the antibody-combining site.

## 4 Conclusions

Binding promiscuity is a characteristic of many proteins and is an essential feature for efficient cellular communication. In the past (Fornili et al., 2013) we characterised the essential role played by promiscuous residues at the surface of multi-binding proteins (hubs) and revealed an important feature the flexibility exerted by these residues and their enhanced motion. This plasticity can be efficiently exploited in supporting diversity of interaction and effective binding. Here we focused on another important class of proteins, antibodies, and highlighted the important role of physico-chemical properties of specific residues at the CDR-H3 in conferring promiscuous behaviour to a set of antibodies tested for multiple binding to a small panel of antigens *in-vitro*. Interestingly, we found that it is not the entire region of the paratope that has to exhibit flexibility to effectively modulate promiscuous binding, but this comes at a compromise between the β-sheet content of the CDR-H3 that can hold in place and protrude out of the antibody combining-site, thereby ‘offering’ to the epitope the crucially binding effective residues that can exert their plastic role and explore multiple binding conditions. In analogy with the previous findings, the promiscuity is relegated to only few versatile and intrinsically flexible residues. These are concentrated at the apex of the CDR-H3 β-sheet and found to contribute to the global motion accounted for by the first and second eigenvectors of the variable framework region. The observed properties were crucially different from the ones measured for a comparable class of antibodies that showed no binding preferences in the same tested conditions.

The findings presented here can have important applications in the design of specific antibodies devoid of promiscuous behaviour, as it is well known that in many circumstances antibodies elicited towards a specific antigen are found to bind to structurally unrelated epitopes (James and Tawfik, 2009). Additionally, the role of specific physicochemical determinants in promiscuous protein activities could pave the way to alternative approaches to directed evolution of novel protein functions (Buchholz and Stewart, 2001).

## Appendix A Supplementary data

Fig. S1 Multiple sequence alignments of (A) the heavy chain and (B) the light chain sequences of the promiscuous and non-promiscuous antibodies. Sequences were aligned using the Simple MSA option in T-Coffee (Di Tommaso et al., 2011, Notredame et al., 2000). Fig. S1

Fig. S2 Average secondary structure probabilities in simulation ensembles for i) promiscuous and ii) non-promiscuous CDR-L3 regions. Predictions were calculated using DSSP. Each antibody is represented by a conformational ensemble of 500 tCONCOORD structures, such that the total number of structures in i) is 2000 (4 antibodies) and in ii) is 3000 (6 antibodies). Fig. S2

Fig. S3 Eigenvalue spectrum for GF1 (red) and GF4 (blue). The contribution (in nm^2^) of the first six eigenvectors to the total global motion of the tCONCOORD ensembles (see Materials and Methods) of GF1 and GF4. Fig. S3

Fig. S4 Porcupine representation of the first and second principal components of the promiscuous and non-promiscuous tCONCOORD ensembles. The red spikes represent the direction and relative magnitude of the motion of each C^α^ atom along the first and second principal components. Fig. S4

Fig. S5 Principal component analysis of CDR-L3 dynamics. The contribution of each atom in the CDR-L3 loop of GF1 (red) and GF4 (blue) to the first and second eigenvectors measured in nm^2^. Fig. S5

Fig. S6 Root mean square deviation of CDR-H3 loops from tCONCOORD trajectories. The spatial distribution of the CDR-H3 loops from an ensemble of 500 structures was measured for the promiscuous (red) and non-promiscuous (blue) sets of antibodies. Fig. S6
