# Supplementary material for: Promiscuous antibodies characterised by their physico-chemical properties: From sequence to structure and back
Source: Prog Biophys Mol Biol. 2017 Sep;128:47–56. doi: 10.1016/j.pbiomolbio.2016.09.002 (PMC6167913; doi:10.1016/j.pbiomolbio.2016.09.002)
Supplement: Fig. S1 — Multiple sequence alignments of (A) the heavy chain and (B) the light chain sequences of the promiscuous and non-promiscuous antibodies. Sequences were aligned using the Simple MSA option in T-Coffee (Di Tommaso et al., 2011, Notredame et al., 2000). [file mmc1.pdf]

A

Promiscuous Heavy chains

```
 *
 BAD AVG GOOD
 *
GF1   :  98
GF5   :  98
GF7   :  98
GF9   :  98
con   :  96

GF1   SLRLSCAASGFTFSSYSMNWVRQAPGKGLEWVAVISYDGSNKYYADSVKGRFTISRDNSKNTLYLQMNSLRAEDT
GF5   SLRLSCAASGFTFSSYAMSWVRQAPGKGLEWVSYISSSGSTIYYADSVKGRFTISRDNSKNTLYLQMNSLRAEDT
GF7   ---LSCAASGFTFSSYGMHWVRQAPGKGLEWVAVIWYDGSNKYYADSVKGRFTISRDNSKNTLYLQMNSLRAEDT
GF9   SLRLSCAASGFTFSSYGMHWVRQAPGKGLEWVAVIWYDGSNKYYADSVKGRFTISRDNSKNTLYLQMNSLRAEDT

con      **************.* **************: *   .**. ***********************************

GF1   AVYYCARVVGSSK----WDHAWYFDLWGRGTLVTVSS
GF5   AVYYCAKASLVRY----FDWLFNFDYWGQGTLVTVSS
GF7   AVYYCARDMVLEW----SYYYYGMDVWGQGTTVTVSS
GF9   AVYYCARGPHRYGDYGGYYYYYGMDVWGQGTTVTVSS

con   ******:          :  :* **:** *****
```

CDR-H3

Non-promiscuous Heavy chains

```
 *
 BAD AVG GOOD
 *
GF2    :  92
GF3    :  96
GF4    :  96
GF6    :  95
GF8    :  96
GF10   :  96
cons   :  94

GF2    ETLSLTCTVSGGSISSSSYYWGWIRQPPGKGLEWIGSIYYSGS-TYYNPSLKSRVTISVDTSKNQFSLKLSSVTA
GF3    -SLRLSCAASGFTF--SSYGMHWVRQAPGKGLEWVSAISGSGGSTYYADSVKGRFTISRDNSKNTLYLQMNSLRA
GF4    -SLRLSCAASGFTF--SSYAMHWVRQAPGKGLEWVAVISYDGSNKYYADSVKGRFTISRDNSKNTLYLQMNSLRA
GF6    -SLRLSCAASGFTF--SSYGMHWVRQAPGKGLEWVAVIWYDGSNKYYADSVKGRFTISRDNSKNTLYLQMNSLRA
GF8    ----LSCAASGFTF--SSYGMHWVRQAPGKGLEWVAVIWYDGSNKYYADSVKGRFTISRDNSKNTLYLQMNSLRA
GF10   -SLRLSCAASGFTF--SSYWMSWVRQAPGKGLEWVANIKQDGSEKYYVDSVKGRFTISRDNAKNSLYLQMNSLRA

cons       *:*:.** ::  ***   *:**.*******:. *   .*.  .**  *:*.*.*** *.:** : *::.*: *

GF2    ADTAVYYCARGRYRDYSNP-------ACVFDYWGQGTLVTVSS
GF3    EDTAVYYCAKDWEKYCSGG-------SCQYDYWGQGTLVTVSS
GF4    EDTAVYYCAKEYLKGRDGY-------NYYFDYWGQGTLVTVSS
GF6    EDTAVYYCARAYNRCSGGSCYEHETTLTGFDYWGQGTLVTVSS
GF8    EDTAVYYCASPTPSGSSARYY----YYYGMDVWGQGTTVTVSS
GF10   EDTAVYYCAREGCGGDCYSYY----YYYYMDVWGKGTTVTVSS

cons    *********                      * **:** *****
```

CDR-H3

B

Promiscuous Light chains

```
*
 BAD AVG GOOD
*
GF1    :   99
GF5    :   99
GF7    :   99
GF9    :   99
con    :   99

GF1     ---TQSPATLSASVGDRVTITCRVSQGISSYLNWYRQKPGKVPKLLIYSASNLQSGVPSRFSGSGSGTDFTLTIS
GF5     ---TQSPATLSASVGDRVTITCRASQGISNYLAWYQQKPGKVPKLLIYAASTLQSGVPSRFSGSGSGTDFTLTIS
GF7     IQLTQSPSSVSASVGDRVTITCRASQGISSWLAWYQQKPGKAPKLLIYAASSLQSGVPSRFSGSGSGTDFTLTIS
GF9     IQMTQSPSSVSASVGDRVTITCRASQGISSWLAWYQQKPGKAPKLLIYAASSLQSGVPSRFSGSGSGTDFTLTIS

con        ****:::*************.*****.:* **:*****.******:**.***********************

GF1     SLQSEDFAVYYCQQYNNW-PRTFGQGTRLEIK
GF5     SLQPEDVATYYCQKYNSAPPFTFGPGTKVDIK
GF7     SLQPEDFATYYCQQANSF-PWTFGQGTKLEIK
GF9     SLQPEDFATYYCQQANSF-PITFGQGTRLEIK

con     ***.**.*.****: *.  * *** **:::**
```

CDR-L3

Non-promiscuous Light chains

```
*
 BAD AVG GOOD
*
GF2     :   88
GF3     :   86
GF4     :   89
GF6     :   89
GF8     :   84
GF10    :   88
cons    :   89

GF2     S-ALTQPPSASGSPGQSVTISCTGTSS-DV----GGYNYVSWYQQHPGKAPKLMIYEVS----KRPSGVPDRFSG
GF3     ---TQSPDSLAVSLGERATINCKSSQS-VLYSSNN-KNYLAWYQQKPGQPPKLLIYWAS----TRESGVPDRFSG
GF4     ---LTQPPSASGSPGQSVTISCTGTSS-DV----GGYNYVSWYQQHPGKAPKLMIYEVS----KRPSGVPDRFSG
GF6     Y-ELTQPPSASGTPGQRVTISCSGSSS-NI----G-SNYVYWYQQLPGTAPKLLIYRNN----QRPSGVPDRFSG
GF8     QSVLTQPASLSASPGASASLTCTLRSGINV----G-TYRIYWYQQKPGSPPQYLLRYKSDSDKQQGSGVPSRFSG
GF10    ---TQSPATLSASVGDRVTITCRASQ---V----I-SNYLAWYQQKPGKVPNLLIYAAS----TLQSGVPSRFSG

cons         .* : : : *  .::.*   .  :      : **** **  *: ::  .        ****.****

GF2     SKS--GNTASLTVSGLQAEDEADYYCSSYAGS---NNLRVFGGGTKLTVL
GF3     SGS--GTDFTLTISSLQAEDVAVYYCQQYYST---PRI-TFGPGTKVDIK
GF4     SKS--GNTASLTVSGLQAEDEADYYCSSYAGS---NNL-EFGGGTKLTVL
GF6     SKS--GTSASLAISGLRSEDEADYYCAAWDDSLSGPSW-VFGGGTKLTVL
GF8     SKDASANAGILLISGLQSEDEADYYCMIWHSS---A-C-VFGGGTKLTVL
GF10    SGS--GTDFTLTISSLQPEDVATYYCQVYNSA---PPV-TFGQGTKVEIN

cons    * .  ..    * :*.*:.** * ***   : .:      ** ***: :
```

CDR-L3
