# Supplementary figures and images for: Promiscuous antibodies characterised by their physico-chemical properties: From sequence to structure and back
Source: Prog Biophys Mol Biol. 2017 Sep;128:47–56. doi: 10.1016/j.pbiomolbio.2016.09.002 (PMC6167913; doi:10.1016/j.pbiomolbio.2016.09.002)

Promiscuous CDR-L3

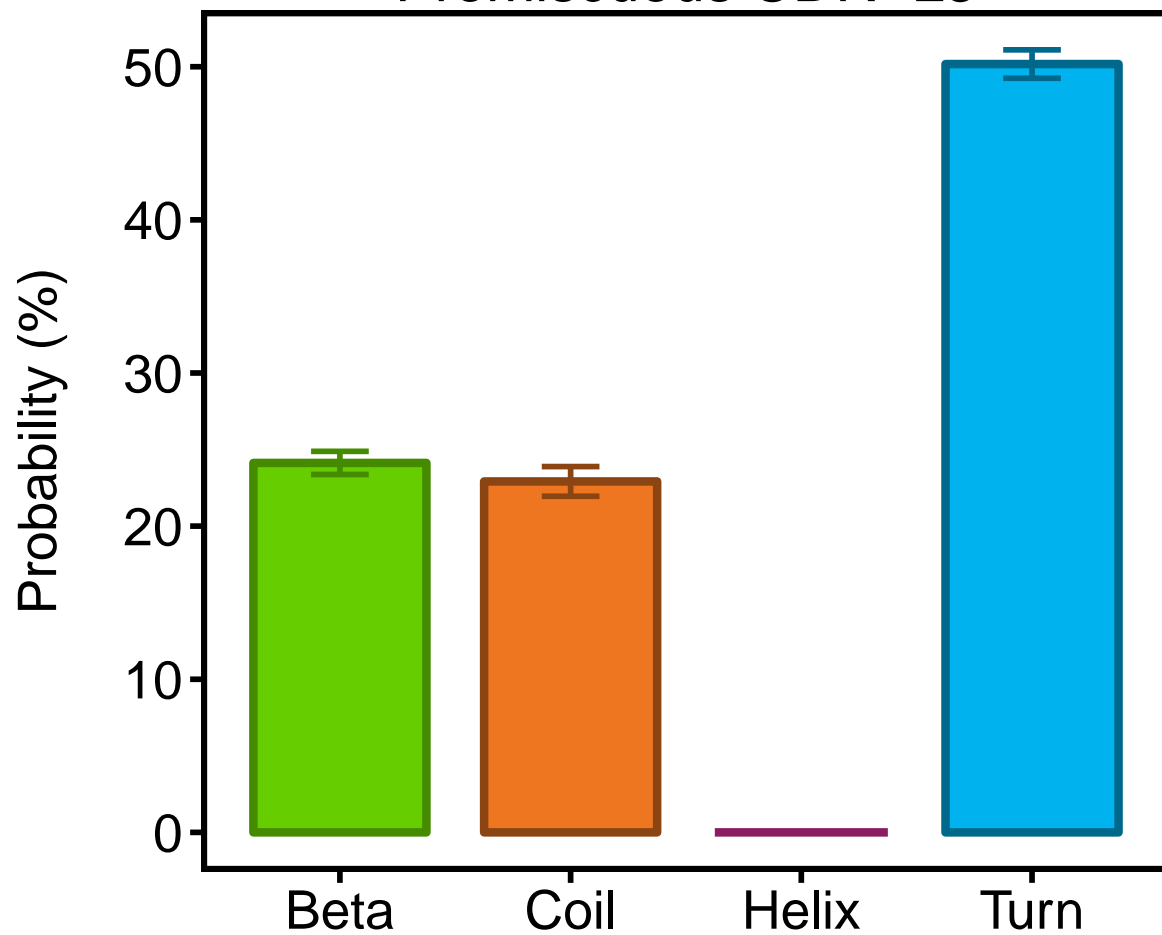

Non-Promiscuous CDR-L3

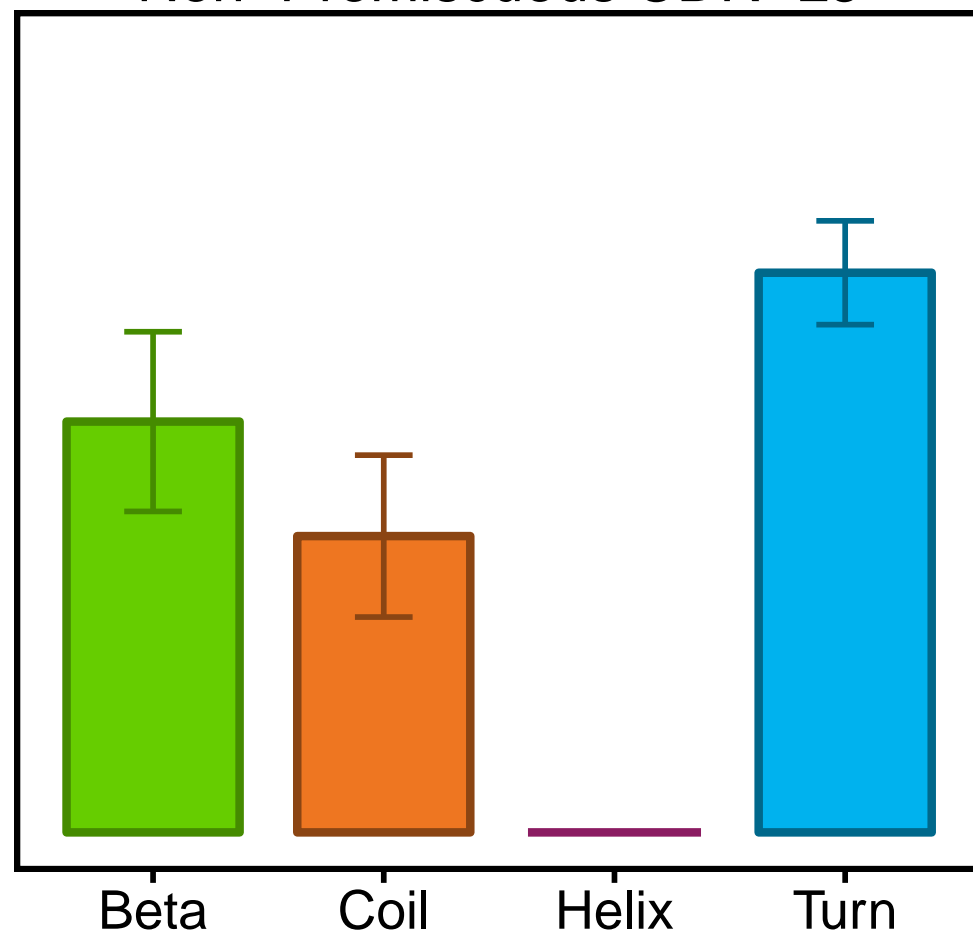

Secondary structure

Supplement: Fig. S2 — Average secondary structure probabilities in simulation ensembles for i) promiscuous and ii) non-promiscuous CDR-L3 regions. Predictions were calculated using DSSP. Each antibody is represented by a conformational ensemble of 500 tCONCOORD structures, such that the total number of structures in i) is 2000 (4 antibodies) and in ii) is 3000 (6 antibodies). [file mmc2.pdf]

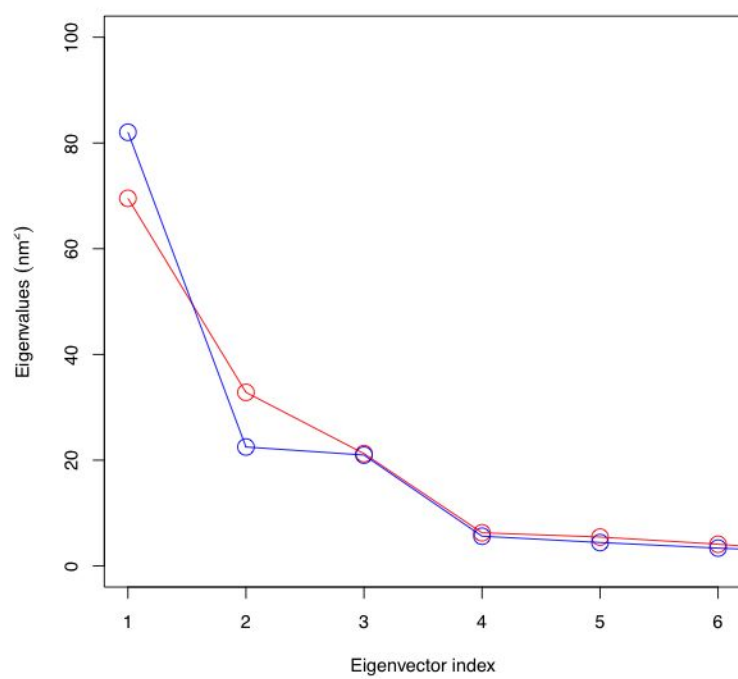

Supplement: Fig. S3 — Eigenvalue spectrum for GF1 (red) and GF4 (blue). The contribution (in nm2) of the first six eigenvectors to the total global motion of the tCONCOORD ensembles (see Materials and Methods) of GF1 and GF4. [file mmc3.pdf]

GF1

GF4

PC1

H3

L3

H3

L3

PC2

H3

L3

H3

L3

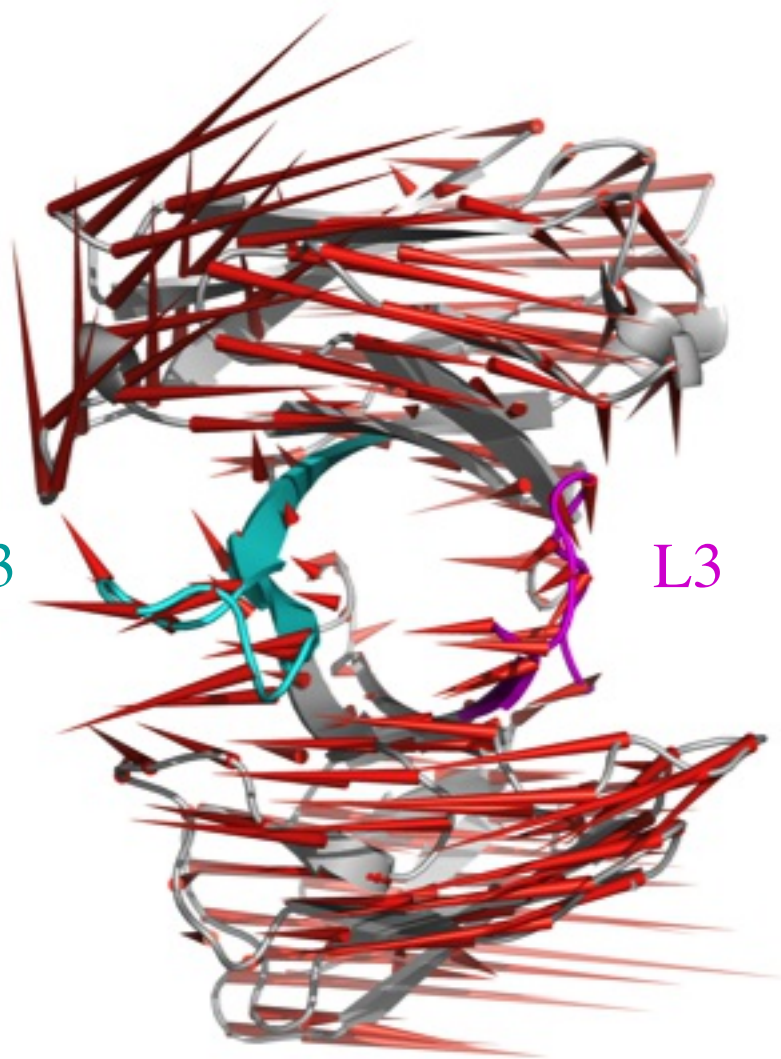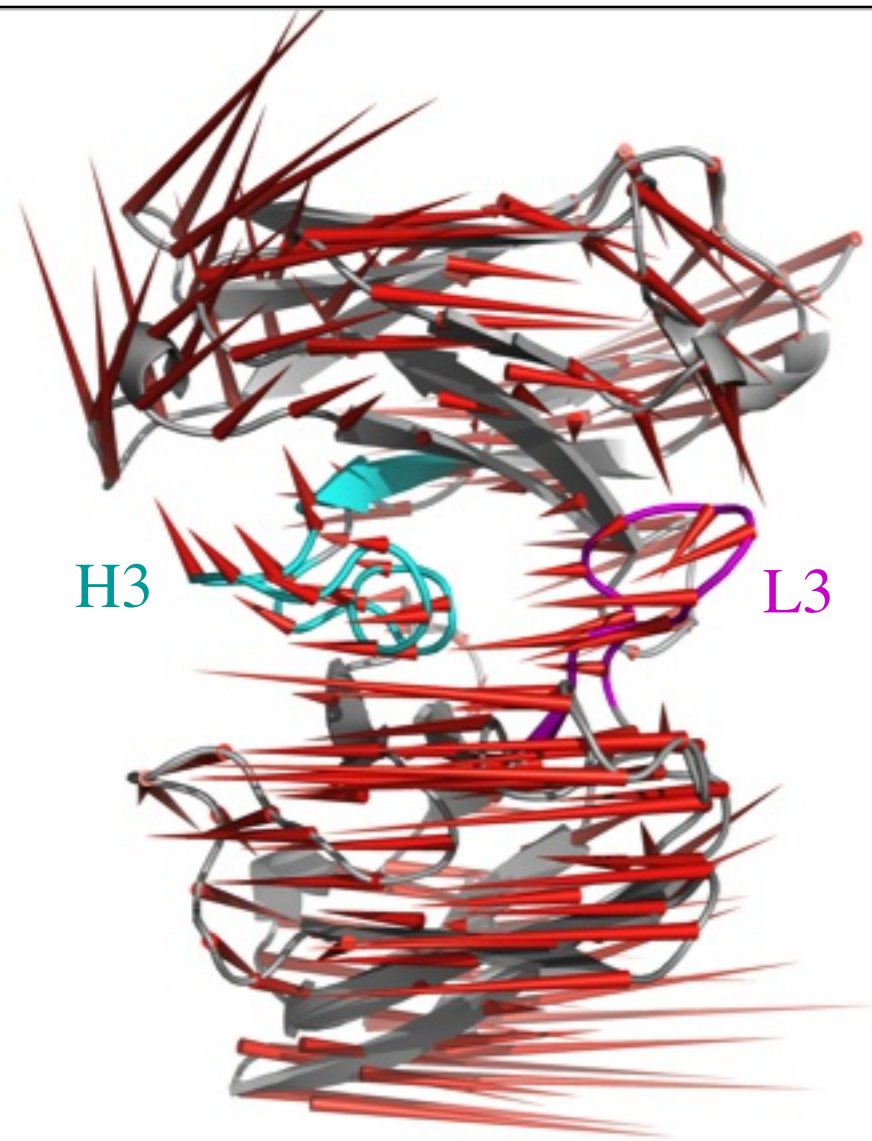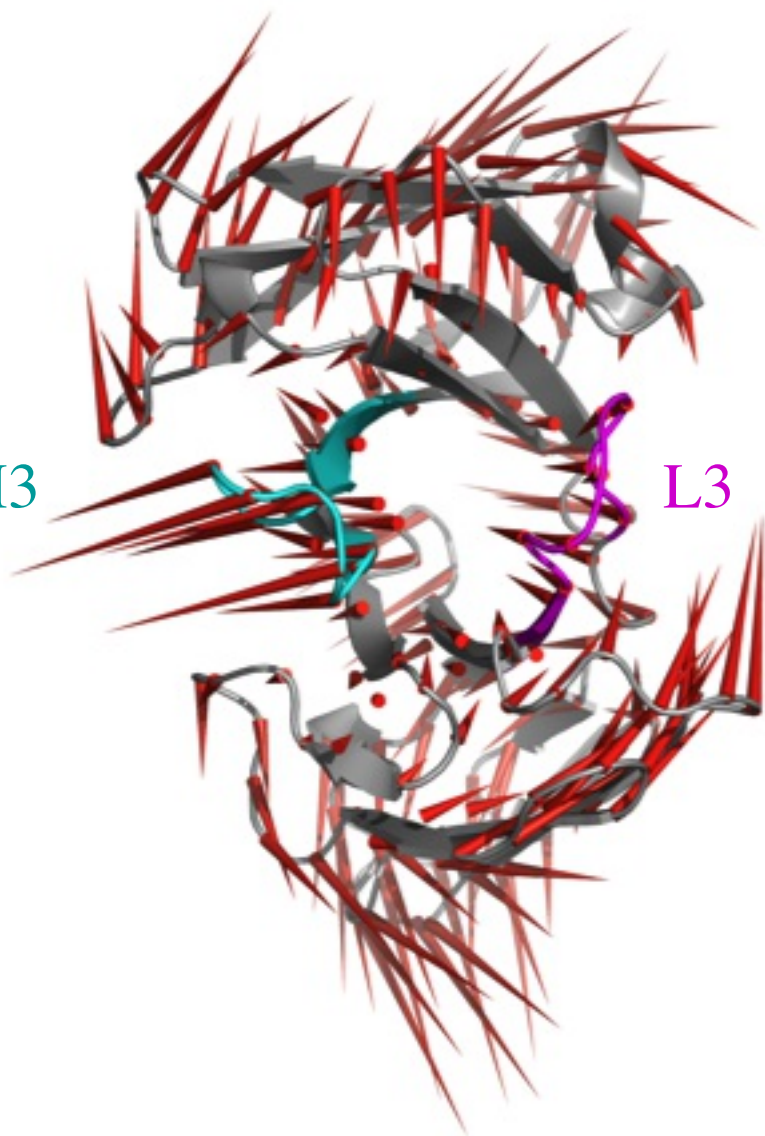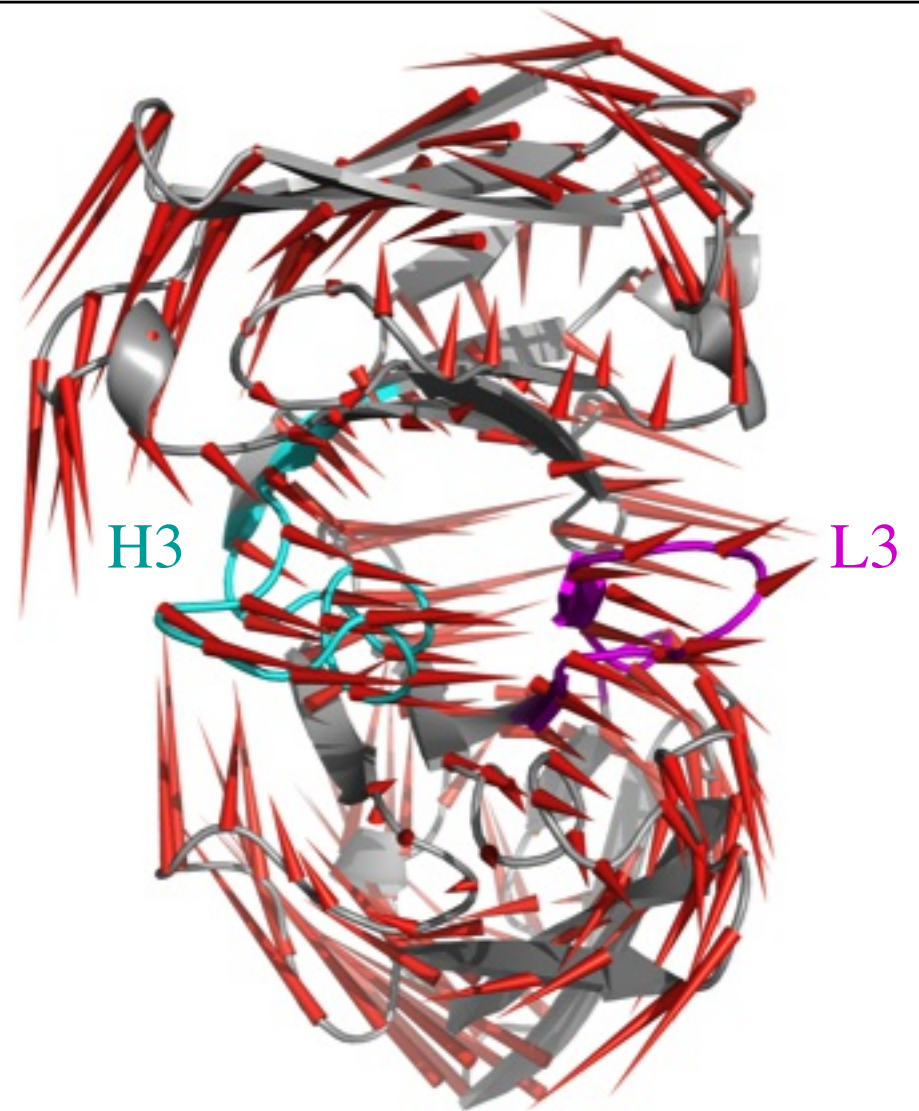

Supplement: Fig. S4 — Porcupine representation of the first and second principal components of the promiscuous and non-promiscuous tCONCOORD ensembles. The red spikes represent the direction and relative magnitude of the motion of each Cα atom along the first and second principal components. [file mmc4.pdf]

**CDR-L3**

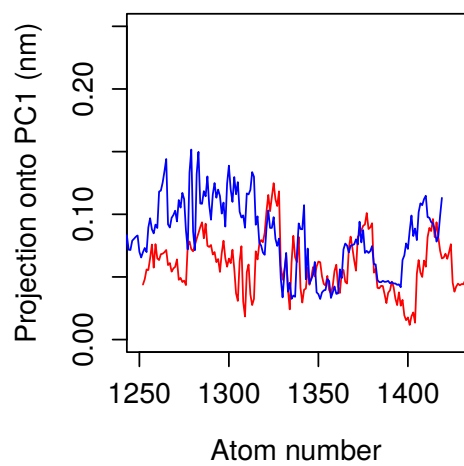

**CDR-L3**

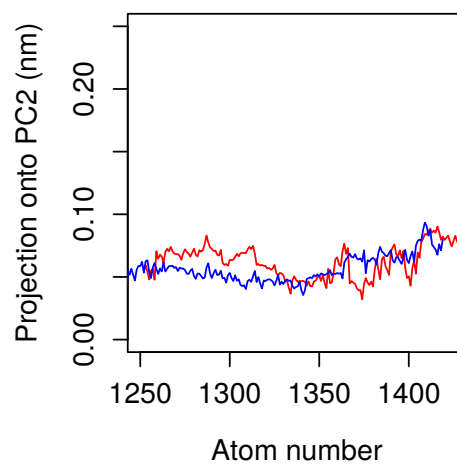

Supplement: Fig. S5 — Principal component analysis of CDR-L3 dynamics. The contribution of each atom in the CDR-L3 loop of GF1 (red) and GF4 (blue) to the first and second eigenvectors measured in nm2. [file mmc5.pdf]

**GF1**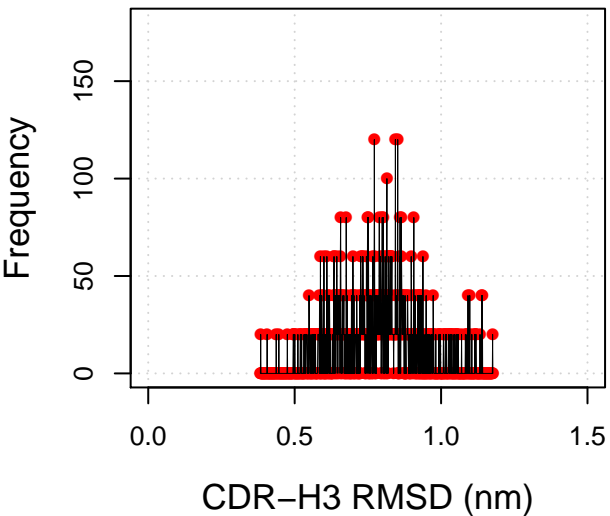**GF5**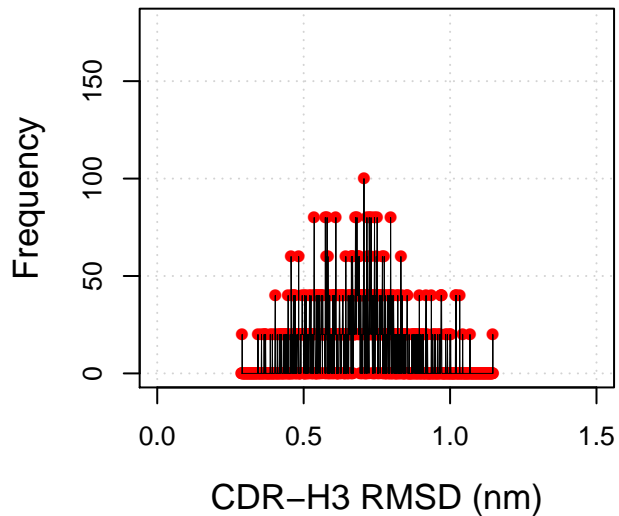**GF7**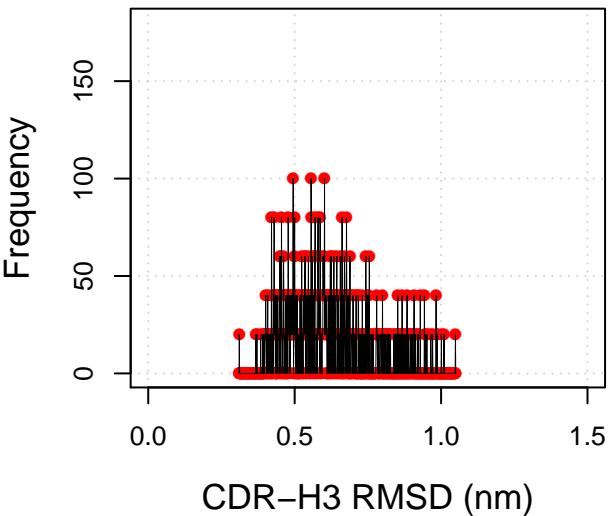**GF9**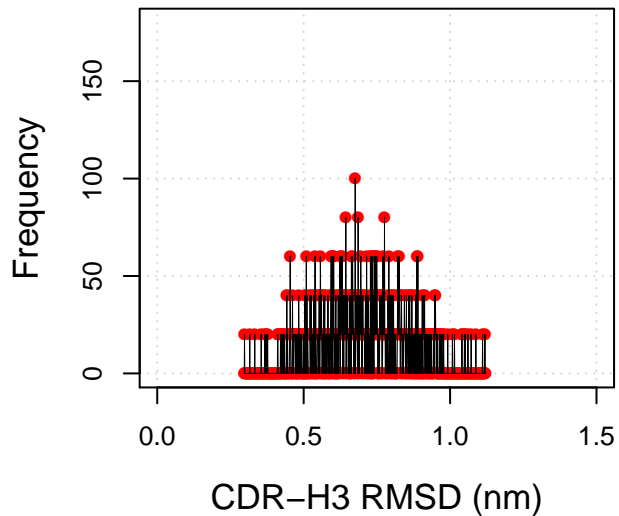

**GF2**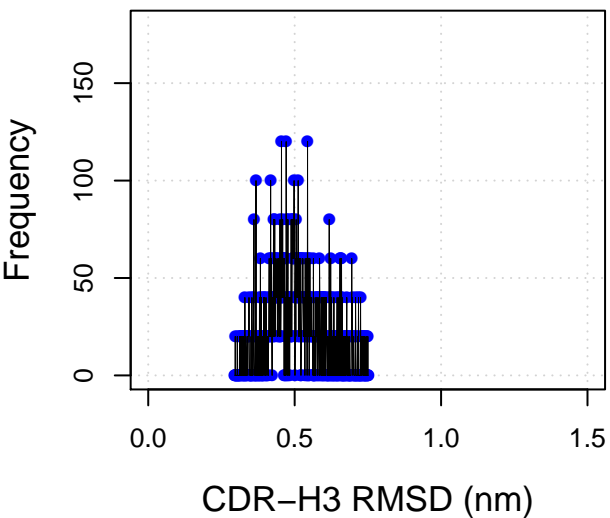**GF3**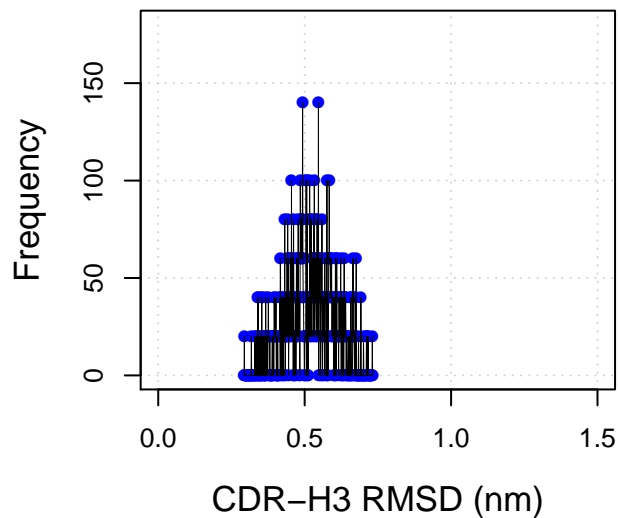**GF4**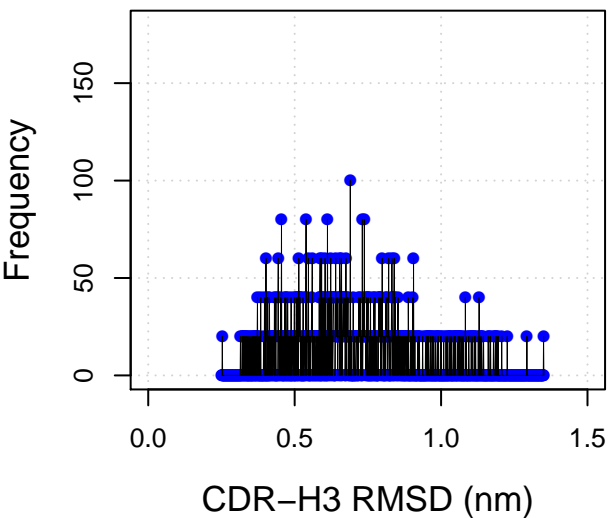**GF6**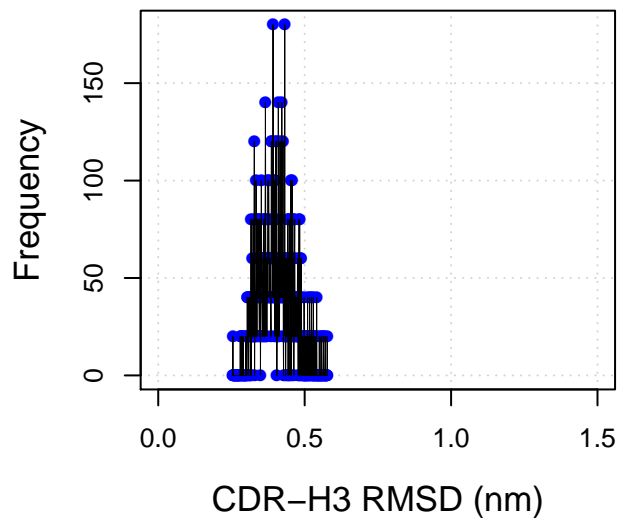

**GF8**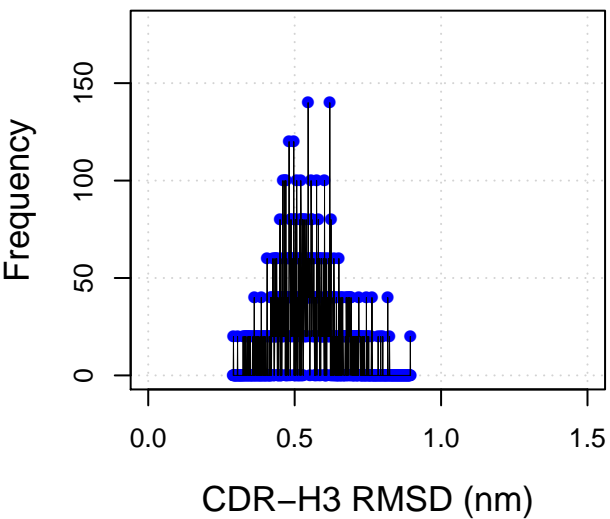**GF10**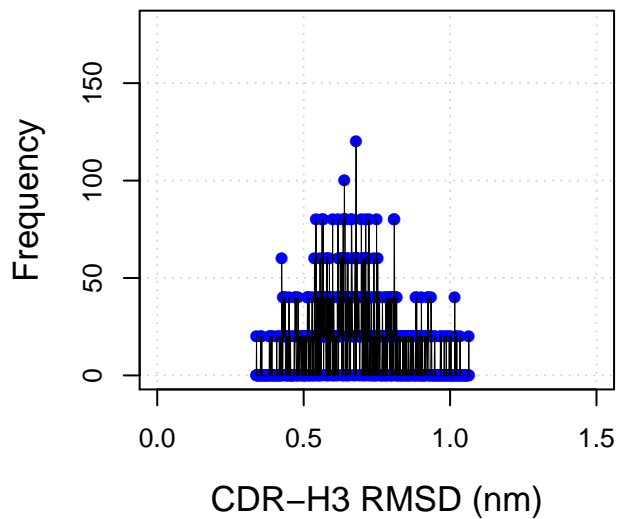

Supplement: Fig. S6 — Root mean square deviation of CDR-H3 loops from tCONCOORD trajectories. The spatial distribution of the CDR-H3 loops from an ensemble of 500 structures was measured for the promiscuous (red) and non-promiscuous (blue) sets of antibodies. [file mmc6.pdf]
