# Supplementary material for: Promiscuous antibodies characterised by their physico-chemical properties: From sequence to structure and back
Source: Prog Biophys Mol Biol. 2017 Sep;128:47–56. doi: 10.1016/j.pbiomolbio.2016.09.002 (PMC6167913; doi:10.1016/j.pbiomolbio.2016.09.002)
Supplement: Supplementary file 7 [file mmc7.docx]

| ID | IGHV | IGHD | IGHJ | CDR-H3 | IGLV | IGLJ | CDR-L3 |
| --- | --- | --- | --- | --- | --- | --- | --- |
| GF1 | IGHV3-30 | IGHD2-15 | IGHJ2 | ARVVGSSKWDHAWYDL | IGKV1-37 | IGKJ5 | QQYNNWPRT |
| GF2 | IGHV4-39 | IGHD4-11 | IGHJ4 | ARGRYRDYSNPACVFDY | IGLV2-8 | IGLJ3 | SSYAGSNNLRV |
| GF3 | IGHV3-23 | IGHD2-15 | IGHJ4 | AKDWEKYCSGGSCQYDY | IGKV4-1 | IGKJ3 | QQYYSTPRIT |
| GF4 | IGHV3-30 | IGHD5-24 | IGHJ4 | AKEYLKGRDGYNYYFDY | IGLV2-8 | IGLJ3 | SSYAGSNNLE |
| GF5 | IGHV3-23 | IGHD3-9 | IGHJ4 | AKASLVRYFDWLFNFDY | IGKV1-27 | IGKJ3 | QKYNSAPPFT |
| GF6 | IGHV3-33 | IGHD2-15 | IGHJ4 | ARAYNRCSGGSCYEHETTLTGFDY | IGLV1-47 | IGLJ3 | AAWDDSLSGPSWV |
| GF7 | IGHV3-33 | IGHD3-3 | IGHJ6 | ARDMVLEWSYYYYGMDV | IGKV1D-12 | IGKJ2 | QQANSFPWT |
| GF8 | IGHV3-30 | IGHD3-10 | IGHJ6 | ASPTPSGSSARYYYYYGMDV | IGLV5-45 | IGLJ3 | MIWHSSACV |
| GF9 | IGHV3-33 | IGHD4-17 | IGHJ6 | ARGPHRYGDYGGYYYYYGMDV | IGKV1D-12 | IGKJ5 | QQANSFPIT |
| GF10 | IGHV3-7 | IGHD2-21 | IGHJ6 | AREGCGGDCYSYYYYYYMDV | IGKV1-27 | IGKJ1 | QVYNSAPPVT |

Table S1. Ten antibody heavy and light chain sequences selected for cloning based on the difference in frequency of their properties.

Table S2. PDB codes for all species and Human datasets.

**658 Ig structures from all species in the PDB**

1iqd 1iqw 4g6f 3fo2 4kvc 3grw 4↵y 3ggw 3zkq 1pz5 1igm 4jr9 4i77 3ifl 1u0q 1nlb 1za6 1q9w 3giz 3fo9 1zan 3kym 3mlr 2osl 4aeh 1opg 1s3k 3k7u 1yej 3inu 4hpy 1dfb 3bn9 1ndg 4hk0 1lo4 1egj 3ux9 3vg9 1sy6 2otu 3eak 2b1h 4dtg 3lrs 3phq 2g5b 3q6g 3sqo 2w60 3tcl 3zkm 1psk 2h9g 1kiq 1mam 4hs8 3m8o 35c8 2vxv 2hwz 3tnn 3utz 3ezj 7fab 1yjd 3efd 4m48 1rih 2cmr 3g5y 1sbs 3k80 3r1g 4jb9 1h8s 3t65 4jo4 4hkx 2zch 3eot 3pp4 1fn4 1ikf 3b9v 1qd0 1fvd 1gig 3r06 1nak 1cly 2qqn 3okd 4ene 2c1p 4hie 2xzc 3go1 2e27 3hc3 43c9 1dl7 4ht1 4at6 3qhz 1uj3 2nr6 1cz8 3t3p 4hc1 4hzl 3l95 3gjf 4jzn 1c5d 4dkf 2adg 2fb4 3auv 1ob1 1yy8 3eba 4ky1 2y6s 1ghf 1f3d 1l7t 4aei 1hil 1a2y 2eh7 4hjj 1igc 2or9 1u6a 3u30 1rhh 1wej 2aju 3ujt 4b41 3nh7 1f8t 2op4 1tqb 1t2j 2x89 3o2d 4g5z 3vfg 4jqi 1ay1 2hfg 1mim 2gsi 4dqo 4fql 1jpt 1mvf 1c5c 4idl 3mck 1nsn 3cfb 4dvb 4jo1 3qxt 1clo 4hxa 3liz 2xzq 3mlw 3tpk 3fzu 1uz8 4fqi 1kcr 1gpo 1ic7 1bz7 4jam 1kb5 4laj 4k3e 2f5a 4↵v 2zpk 2vl5 4amk 2vxq 2fat 1rz7 1mex 3bkj 1ors 1a6v 1sm3 3i02 4gsd 3fn0 3s96 1for 1fh5 3mbx 3pnw 3vi3 1dvf 1ai1 1p7k 1ri8 1n4x 3zkx 4fqq 1kxt 4fz8 2agj 1jfq 3juy 1kxq 2yk1 3g04 3eyf 3b9k 1mnu 3i9g 3u9p 2bdn 1eap 1flr 1mh5 3r0m 4k3j 3qeh 2qqk 1tzi 2q8b 4gag 3dv6 3d85 3gm0 2a9m 3g9a 3s35 4h0i 4jha 2d7t 2ipu 4eig 1mj8 3u7y 4kuc 2z92 3upc 2w9d 1rur 1aqk 1fai 3qpq 3gkw 3sob 2vq1 3na9 2g75 3u1s 1q72 1zea 1frg 1kcv 1ol0 3se8 1xgy 2brr 1igf 4jm4 2o5x 3ifp 4hwe 1jgl 1rjl 1plg 3vw3 1dn0 2jel 3e8u 3lh2 1mqk 3idx 4gmt 2xt1 2g60 3i75 1nj9 1etz 2r0l 3tnm 2hrp 4gq9 1mju 3idg 3d9a 1dzb 1h8n 4jg1 4eow 4jn2 4hs6 4krn 2j4w 2xkn 3qsk 3o6k 3mcl 4fqj 3pgf 2aab 2hh0 3k74 2v7h 3sdy 3b2u 3ijh 3ujj 3ojd 1nbv 1um5 1j05 4hix 1fl5 3uc0 3lex 12e9 1e6o 2xa8 1ind 3u2s 3ls5 1ct8 1jgu 1t4k 1ce1 1jn6 3v6o 1ejo 2uzi 4gft 1jrh 3dvg 3k2u 3mxw 3skj 1n0x 1keg 4j6r 4dka 4leo 2wzp 3iet 3cvi 1qfu 1il1 1nc2 3uji 2vxs 2arj 4fze 4kph 4krm 1mfa 1zv5 1i7z 4ers 3oaz 3ln9 1yc7 4lst 4d9l 1uwe 2xqb 3↵d 1fj1 4f33 1aif 4aq1 3cx5 1pg7 1vge 2dqu 1f4w 4jpk 1kel 4fnl 4gxv 2hmi 3hnt 3ra7 3u0t 4k2u 1cic 3hi6 4jpw 1c12 4m43 1igt 1t3f 3qot 3eo9 2jb5 4k3d 3i2c 4gw4 3so3 3cfd 2xa3 4g3y 4fhb 3dur 2ih3 2bmk 1zvy 4h0h 2aep 2pcp 3ks0 2p45 2cju 4krp 4k7p 1kfa 3rvv 1nl0 2a6i 1ngz 3eo0 1rmf 3stb 4h20 1cr9 3qg6 1ztx 1dee 3bky 3oz9 2zkh 1a6t 1kxv 1fns 2ck0 3h42 3kr3 1hi6 2x1q 3ncj 3o0r 1fe8 1qkz 1ad9 1mvu 1mcp 1q0x 2qhr 3ma9 1tzh 1lk3 2vyr 1yee 1ap2 1baf 3umt 3rkd 1y0l 1f58 1mlb 1dqd 4ebq 4ag4 3uls 2uyl 3h3b 2g2r 1sjv 2yc1 3sge 1a4j 1dlf 3cmo 4b5e 3esu 2ok0 4hxb 1ndm 4imk 3cfi 4lsu 1osp 3nfs 1a3r 4lkc 1hcv 3mo1 2adf 2iq9 2wuc 8fab 4jy5 2gcy 1mrd 1f2x 1e4x 3qyc 1dsf 3c08 3v0w 2h1p 1g9m 2v7n 3ldb 3clf 1jhl 1eo8 1bj1 1fsk 1ncw 3hc4 1kcu 2uud 3s34 1igj 1uwx 2ddq 1lo0 3gk8 4hpo 4f57 1dqq 2j88 3gkz 2xra 3mly 1x9q 4j8r 4hlz 4hcr 3se9 3ntc 3hr5 3o2v 3qcu 3qpx 3q3g 3dif 3hzm 4m61 2hkf 3dsf 1l7i 3lmj 2qsc 1bfv 1t66 4dcq 1ohq 4i9w 4d9q 3v0a 1ngy 4ej1 2ypv 4hbc 3v6f 3u0w 1nld 3esv 4hfw 4jy6 3p9w 2fx7 3h0t 2vxt 1h0d 2r8s 4lmq 1ktr 3ifo 1ggc 2xtj 1dql 4dgy 4g6m 2ny1 2ghw 1emt 3qwo 2gki 1d5i 1ibg 4lsp 3gi9 2x1o 3uyp 3k1k 2xqy 3mj8 1uyw 4fqc 3kdm 3nzh 3nps 1xiw 1i9j 2xwt 3v4u 1c1e 4h88 2oz4 3p0y 1jv5 1gaf 3l5y 3g6a 1wt5 4m1d 1fgn 2r56 1p2c 1oaq 3nz8 1dbb 1p4b 3u6r 1nmb 4jdv 4fq2 3dgg 3ghe 3gnm 1seq 3n9g 2v17 2fbj 2x1p 1bln 1nfd 4al8 1sjx 1ynl 1h3p 1iai 1yqv 2p4a 4jpi 1w72 1nca 2ai0 3gbn 1t2q 3qq9 2aj3 1hq4

**248 Ig structures from the Human PDB**

1iqd 3u0w 1it9 1n0x 4g6f 4j6r 4fqq 1uwe 3lh2 3grw 2xra 3mbx 3hi6 3hc4 3mlw 2vxs 3fzu 4fqi 3s34 4jpi 1za6 3giz 4gw4 1i7z 3kym 4jam 3mlr 4hpo 2osl 4f57 1opg 2f5a 1s3k 3inu 4hpy 1dfb 1t2j 4d9l 1ad0 2xqb 3bn9 3mly 1x9q 1rz7 4imk 3aaz 3ux9 4hcr 3se9 3ntc 4gsd 3o2v 3fn0 2b1h 4dtg 3lrs 1vge 3qpx 3dvg 1fh5 3qcu 3tcl 3dif 3zkm 4gxv 3upc 1l7i 3nps 3lmj 4ky1 2qsc 3u0t 4fnl 4fz8 3r1g 2agj 3juy 2vxv 1ohq 3tnn 4jpw 1y0l 1ngy 7fab 3g04 3eyf 3qot 3eo9 2jb5 3fo2 4fql 4hfw 2fx7 4k7p 3h0t 1h0d 4lmq 4g3y 2h9g 3uji 2xtj 4jb9 3tnm 3sqo 1igm 1dql 2qqk 3qeh 3p9w 4dgy 4g6m 2ny1 3d85 2xzc 2a9m 4krp 4hkx 4fze 4dkf 3s35 1ikf 1d5i 4jha 2d7t 3b9v 1fvd 3u7y 1ngz 4lsp 3idx 1aqk 4g6a 3gkw 3h42 2qqn 3sob 3na9 2g75 3u1s 3bky 2zkh 4jy6 1ol0 4fqc 4hie 3kdm 3nzh 3qpq 3go1 4hs8 3kr3 3hc3 2xwt 2ghw 4jm4 2o5x 3se8 3uls 1uj3 4hwe 3ma9 3ncj 1dn0 3p0y 1jv5 1gaf 2vyr 4ers 3l5y 3g6a 1wt5 3gjf 4jzn 4m1d 4lst 2aj3 1nl0 2r56 3m8o 3dgg 2hfg 2fb4 3auv 2cmr 3so3 1yy8 3oaz 1dee 3u6r 1cly 3idg 2eiz 4jdv 4fq2 3qhz 1bvk 4eow 1ad9 2yc1 4hjj 3ghe 4hs6 3n9g 4al8 4i77 3mcl 3pgf 1u6a 3u30 1rhh 2hh0 3t2n 3sdy 2uzi 3b2u 3nh7 4lsu 3ujj 3nfs 4d9q 4lkc 1fl5 2x89 3mo1 2yk1 2xa8 3u2s 4g5z 1w72 2wuc 8fab 4jy5 4fqj 3gbn 2vxq 1mim 1t3f 3qyc 4dqo 3c08 3qq9 1jpt 1g9m 1c5c 3k2u 3mxw 3skj 4hk0
